# Luteolin-Fabricated ZnO Nanostructures Showed PLK-1 Mediated Anti-Breast Cancer Activity

**DOI:** 10.3390/biom11030385

**Published:** 2021-03-05

**Authors:** Shiva Prasad Kollur, Shashanka K. Prasad, Sushma Pradeep, Ravindra Veerapur, Sharanagouda S. Patil, Raghavendra G. Amachawadi, Rajendra Prasad S, Ghada Lamraoui, Abdulaziz A. Al-Kheraif, Abdallah M. Elgorban, Asad Syed, Chandan Shivamallu

**Affiliations:** 1 Department of Sciences, Amrita School of Arts and Sciences, Amrita Vishwa Vidyapeetham, Mysuru Campus, Mysuru, Karnataka 570 026, India; 2 Department of Biotechnology and Bioinformatics, School of Life Sciences, JSS Academy of Higher Education and Research, Mysuru, Karnataka 570 015, India; shashankaprasad@jssuni.edu.in (S.K.P.); sushmap@jssuni.edu.in (S.P.); 3 Department of Metallurgy and Materials Engineering, Malawi Institute of Technology, Malawi University of Science and Technology, P.O. Box 5916, Limbe 312229, Malawi; rveerapur@must.ac.mw; 4 ICAR-National Institute of Veterinary Epidemiology and Disease Informatics, Yelahanka, Bengaluru, Karnataka 560 064, India; sharanspin13@gmail.com; 5 Department of Clinical Sciences, College of Veterinary Medicine, Kansas State University, Manhattan, KS 66506-5606, USA; agraghav@vet.k-state.edu; 6 Department of Chemistry, Davangere University, Shivagangotri, Davangere, Karnataka 577 007, India; raju.rajendraprasad693@gmail.com; 7 Nature and Life Sciences, Earth and Universe Sciences, University of Tlemcen, Tlemcen 13000, Algeria; lamraouig@gmail.com; 8 Dental Biomaterials Research Chair, Dental Health Department, College of Applied Medical Sciences, King Saud University, P.O. Box 10219, Riyadh 11433, Saudi Arabia; aalkhuraif@ksu.edu.sa; 9 Department of Botany and Microbiology, College of Science, King Saud University, P.O. Box 2455, Riyadh 11451, Saudi Arabia; aelgorban@ksu.edu.sa

**Keywords:** luteolin, ZnONPs, in silico analysis, PLK1 proteins

## Abstract

The present work describes a facile and convenient procedure for synthesizing zinc oxide nanoparticles using luteolin isolated from *Eclipta alba* plant (L-ZnONPs) at room temperature. The formation of as-grown L-ZnONPs was confirmed by X-ray diffraction analysis (XRD), scanning electron microscopy (SEM), transmission electron microscopy (TEM), high-resolution transmission electron microscopy (HR-TEM), and selected area electron diffraction (SAED). The Wurtzite structure of ZnO was observed by its hexagonal phases in diffraction patterns. The SEM images revealed the different sizes and morphologies of L-ZnONPs, with diameters between 12 and 25 nm. The HR-TEM result showed that the inter-planar distance between two lattice fringes was 0.262 nm, which coincides with the d-spacing of (002) and (101) lattice planes of the as-obtained material. The anticancer activity of L-ZnONPs against the breast cancer cell line MCF-7 was greater as compared to that of luteolin or ZnO alone. The mechanistic evaluation of such an activity carried out using in silico methods suggested that the anti-breast cancer activity of L-ZnONPs was mediated by polo-like kinase 1 (PLK1) proteins.

## 1. Introduction

Recent advances in the field of nanoscience and nanotechnology, with a particular aptitude for the preparation of highly ordered nanoparticulates of all types of morphologies, have led to the development of novel materials at the nanoscale level. The large realm in the field of nanoscience lies in the fact that nanoparticles deliver desirable properties and have wide applications in highly functional and effective therapeutic, catalytic, sensing, and photoelectronic devices [1,2,3,4,5,6]. Among metal oxide nanoparticles, zinc oxide is interesting due to its enormous range of applications in various areas such as the medical, optical, magnetic, and gas sensing fields. In addition to these properties, the ZnO nanostructure demonstrates high catalytic efficiency and strong adsorption ability, and is used routinely in the manufacture of sunscreens [7], in ceramics and rubber processing, in wastewater treatment, and as an antimicrobial agent [8,9].

The development of various processes for the synthesis of nano- and micro-scaled inorganic materials has contributed to an understanding of a relatively new and largely unexplored area of research based on the biosynthesis of nanomaterials. Plant extracts possessing metal-interacting multi-functional groups such as hydroxyl, carboxyl, and hetero-aromatic rings offer an excellent opportunity to develop eco-friendly and cost-effective nanostructures that exhibit enhanced biological significance [10,11,12]. Biological applications of green synthesized ZnONPs have gained ample interest in the present scientific scenario [13,14]. Although there are many reports explaining the extraction and isolation of phytomolecules from *Eclipta alba (E. alba)* and their biological potencies [15,16,17,18], no work has been reported on the use of these phytomolecules in capping the metal nanoparticles. In this study, we aimed to synthesize ZnO nanoparticles using a phytomolecule, luteolin, isolated from *E. alba*. The above zinc oxide nanoparticles synthesized using luteolin isolated from *Eclipta alba* (L-ZnONPs) were further screened for their tumoricidal efficacy.

Reports suggest the antioxidant, anti-inflammatory, and anticancer potential of luteolin [19,20,21]. Studies indicate that the flavonoid inhibits tumor proliferation, progression, angiogenesis, and metastasis [22]. Recent investigations endorse the cytotoxic behavior of luteolin across the varieties of cancer [23,24,25,26,27,28,29,30]. With particular reference to breast cancer, Sato et al. (2015) concluded that luteolin demonstrated a biphasic cytotoxic effect on the MCF-7 cell line, meaning that the compound showed greater cytotoxicity at higher concentrations as compared to lower concentrations [31]. Additionally, luteolin, when used at low concentrations, was found to attenuate the cytotoxicity of doxorubicin and increase Bcl-2 protein levels in MCF-7 cells even in the presence of an estrogen receptor antagonist [31]. However, various conducted studies have indicated that luteolin shows potent cytotoxicity against cell lines of various sub-types of breast cancer. Studies have indicated that the compound induces apoptosis in the breast cancer cell lines MCF-7 and MDA-MB-231 via cell cycle arrest in the G_2_/M and S phases [30,31,32,33,34,35]. Anti-breast cancer studies conducted using pertinent cell lines relevant to the molecular subtypes of the cancer showed that luteolin demonstrates cytotoxicity in a dose- and time- dependent fashion by effectively blocking the proliferation of Era-positive MCF-7, an IGF-1-stimulated luminal A subtype, and triple negative/basal-like Era-negative MDA-MB-231 cells [30,31,32,33,34,35,36]. Luteolin has also exhibited potent anti-angiogenic properties in CAM assays [32,33,34,35,36,37]. In addition, luteolin has been concluded to efficiently suppress tumor cell migration and metastatic invasion [37,38].

**Figure 1:**
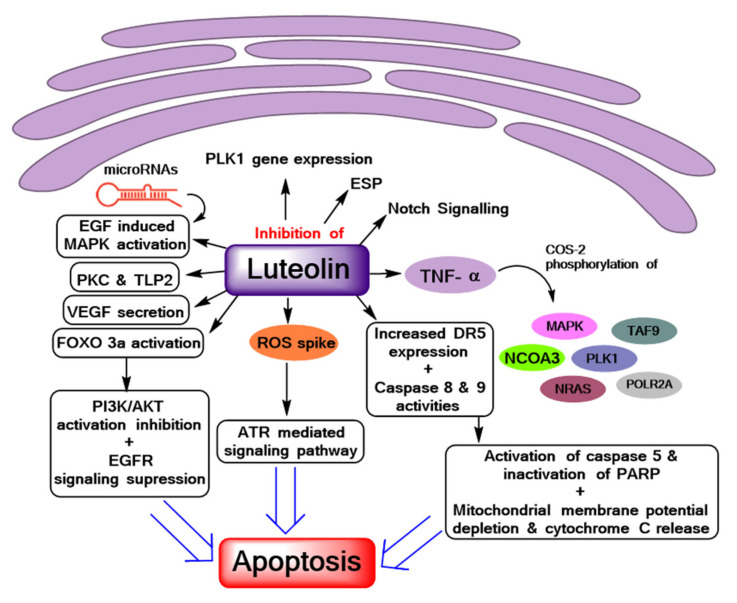
Reported mechanistic bases of luteolin anti-cancer activity. PARP: poly ADP-ribose polymerase; EGFR: endothelial growth factor receptor; ROS: reactive oxygen species; DR5: death receptor 5; PLK1: polo-like kinase 1; PI3K: phosphatidylinositol 3-kinase; MAPK: mitogen-activated protein kinase.

Mechanistic elucidation of luteolin cytotoxicity in breast cancer has been very well established. Luteolin has been reported to demonstrate genotoxic effects due to reactive oxygen species (ROS) generation and the subsequent activation of the ataxia telangiectasia and Rad3-related protein-mediated signaling pathway, resulting in apoptosis occurring in synchrony with nuclear factor kappa B (NF-kB) inhibition, p38 pathway activation, and anti-apoptotic protein depletion [39]. Park et al. (2014) suggested that luteolin promoted apoptosis by enhancing death receptor 5 (DR5) expression and caspase-8 and -9 activities, resulting in the activation of caspase-3 and the inactivation of poly ADP-ribose polymerase (PARP), coupled with mitochondrial membrane potential depletion, cytochrome C release, and up-regulation of Bax expression [40]. It was found to induce apoptosis by FOXO3a activation promotion and phosphatidylinositol 3-kinase (PI3K)/Akt activation inhibition along with suppression of the endothelial growth factor receptor (EGFR) signaling pathway in both MCF-7 and MDA-MB-231 cells [35,41] (Figure 1).

In addition, luteolin has been mechanistically found to inhibit EGF-induced mitogen-activated protein kinase (MAPK) activation, PLK1 gene expression [41,42], the estrogen signaling pathway (by regulation of genes such as TAF9, POLR2A, NCOA3, NRAS, DDX5, NRIP1, NCOR1, and GTF2H2 through the epigenetic mechanism [42,43]), notch signaling and regulation of associated miRNAs, and tumor necrosis factor alpha (TNFa)-induced COS-2 expression, as well as phosphorylation of MAPK/ERK kinase 1/ERK/p90RSK, Akt/p70S6K, MAPK kinase 4/Jun N-terminal kinase (c-JNK)/c-Jun, protein kinase C (PKC), and tumor progression locus 2 (TLP2), in addition to vascular endothelial growth factor (VEGF) secretion in MCF-7 and MDA-MB-231 [44,45,46,47] (Figure 1). Furthermore, when treated on cells growing in hypoxic conditions, luteolin has been established as a chemo-sensitizer to therapeutic drugs for breast cancer [45,46,47,48,49,50]. Notwithstanding, reports also suggest that luteolin in low doses demonstrates cytoprotective behavior against therapeutic drugs [31], implying that the flavonoid demonstrates strictly dose-dependent cytotoxicity and chemo-sensitizing potential.

Furthermore, L-ZnONPs have shown very effective inhibition of several genes involved in breast cancer signaling pathways. Moreover, L-ZnONP–protein interactions have been analyzed using in silico molecular docking approaches. Molecular interaction studies reveal the ability of the ligand/small molecule to bind to the specific protein by forming hydrophobic and non-hydrophobic bonds, thus modifying its expressions and functions.

Meanwhile, zinc oxide (ZnO) nanoparticles, which represent a versatile drug delivery tool, have recently been reported to possess significant tumoricidal activity via ROS generation or the caspase-8 and p53 pathway [51,52,53,54]. However, a better understanding of the mechanistic mode and the resultant cellular consequences is essential. Although the metal oxide has been considered by the US FDA to be a “generally recognized as safe” (GRAS) substance [55], this categorization typically applies to substances that are larger than a micron. Hence, it may be deemed necessary to evaluate the cytotoxicity of the same in both in vitro and in vivo systems.

## 2. Materials and Methods

The precursors, anhydrous zinc acetate (Zn(Oac)_2_) and pristine ZnONPs, were obtained from S.D. Fine Chemicals Ltd. (Mumbai, India), while ethanol and acetone were purchased from Merck Chemical Suppliers (Pune, India). Deionized water collected from an ELGA RO water purifier was used throughout the experiments (Elga Veolia, Lane End, UK). Powder XRD values were recorded on Bruker X-ray diffractometer with a scan range of 20–80° at a 2°/min scan rate using Cu Kα (1.5406 Å) radiation (Bruker, Karlsruhe, Germany). Scanning electron microscopy (SEM) and X-ray mapping images were recorded on a Zeiss microscope (Carl Zeiss, White Plains, NY, USA). Transmission electron microscopy (TEM) images and SAED patterns were recorded on a JEOL 2100F FEG apparatus operating at 200 kV after casting a drop of L-ZnONP for dispersion in ethanol over a Cu grid (Jeol, Akishima, Tokyo, Japan). The ^1^H-NMR spectrum was recorded on a Bruker AC (300 MHz, Yokohama, Japan) spectrometer using tetramethylsilane (TMS) as an internal standard in DMSO-d_6_ solvent. Chemical shifts (δ) are expressed in ppm. Mass spectra were recorded on a Waters SYNAPT G2 mass spectrometer (Malvern, UK) using electrospray ionization (ESI-TOF) operating at an ionization potential of 70 eV.

### 2.1. Plant Material Collection and Extraction of Eclipta alba Phytochemicals

Whole plants of *E. alba* were collected from Srirangapatna, Karnataka, India, (Geographical coordinates: 12.4237° N, 76.6829° E) from May to September 2019. Plant identification was unambiguously performed, and a voucher specimen (No. FLSDWH201) was deposited at the herbarium at the Department of Water and Health, JSS Academy of Higher Education and Research (Mysuru, India). The samples were shade-dried, homogenized using a mixer, and subjected to extraction using various solvents. Fifty grams of the coarse powder of the plant were subjected to hot solvent extraction using methanol (99%). The resulting filtrate was concentrated under a vacuum using a rotary evaporator (Rotavapor R-200, Buchi, Geneva, Switzerland), and the yield of methanol extract was recorded. The extract was further subjected to phytochemical screening in order to evaluate the phyto-constituents based on standard protocols.

### 2.2. Isolation of Bioactive Compound

The above residue (16 g) was suspended in chloroform and then extracted thrice with the same solvent. The chloroform soluble fraction (CSF) was purified through column chromatography using silica gel at an elution rate of 2 mL/min flow with a total elution of 200 mL and a gradient of chloroform:methanol (0.9:0.1) to acquire the fractions CSF1 (0.7 g), CSF2 (0.5 g), CSF3 (0.3 g), CSF4 (0.5 g), CSF5 (0.2 g), CSF6 (0.5 g), CSF7 (0.9 g), CSF8 (1.1 g), and CSF9 (1.0 g). Subsequently, CSF8 was yet again subjected to silica gel column chromatography using a column measuring 50 cm in length and 3 cm in diameter, with the elution rate adjusted to 1 mL/min. The total elution carried out was for 100 mL using the linear gradients of chloroform:acetone (90:10; 80:20; 70:30; 60:40; 50:50; 20:80; *v*/*v*), to obtain 6 sub-fractions. Sub-fraction 3 was further separated by silica gel CC using chloroform:acetone (70:30) followed by re-chromatography on a Sephadex LH-20 column (Sigma-Aldrich, Texas, USA) with methanol as the eluting solvent, yielding luteolin (332.1 mg). The structure elucidation of the luteolin by ^1^H NMR and mass spectral techniques is shown in Figures S1 and S2.

### 2.3. Preparation of L-ZnONPs

**Figure 2:**
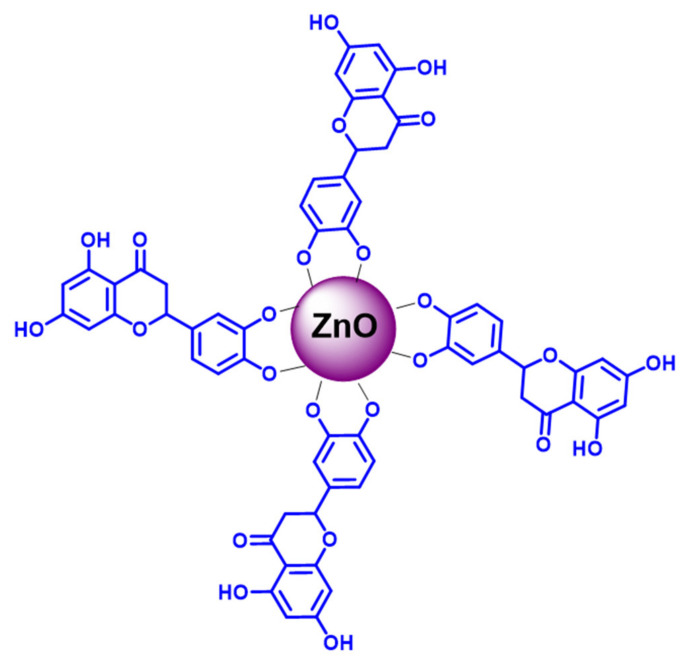
Tentative representation of as-obtained luteolin-functionalized ZnO nanoparticles (L-ZnONPs).

The synthesis of L-ZnONPs was carried out according to the procedure mentioned previously [56]. An aqueous solution of Zn(Oac)_2_ (0.115 g in 10 mL) and luteolin (0.069 g in 10 mL water) was mixed and stirred for 3 h at room temperature (a pH of 8.5 was maintained during synthesis using sodium bicarbonate). The white precipitate formed was filtered off using Whatmann No.1 filter (Analytics, Mumbai, India) paper and washed with ethanol (×5 times) in order to remove the adhered impurities. The above sample was then subjected to calcination in a preheated furnace at 400 °C for 3 h and used for further studies. We could reproduce the experimental results through the aforementioned synthetic procedure. The formation of L-ZnONPs was via 2,3-dihydoxyl groups present in the luteolin molecule, which were oxidized by the reducing the zinc ions to ZnONPs with their electron-donating abilities. The as-obtained luteolin-decorated ZnO nanostructures are depicted in Figure 2. The FT-IR and UV-Visible spectra of the as-obtained L-ZnONPs are depicted in Figures S3 and S4.

### 2.4. Determination of Anticancer Activity of As-Synthesized L-ZnONPs

The cytotoxic effects of luteolin, ZnO nanoparticles, and L-ZnONPs were determined using 3-(4,5-dimethylthiazol-2-yl)-2,5-diphenyltetrazolium bromide, commonly known as MTT. Breast cancer cells (MCF-7) were procured from the ATCC and cultured in Dulbecco’s Modified Eagle Medium (DMEM) with 10% fetal bovine serum (FBS), penicillin (100 IU/mL), and streptomycin (100 µg/mL) in 5% CO_2_ at 37 °C until confluence. The cells were trypsinized using 0.05% trypsin-EDTA solution and checked for viability using a hemocytometer. One hundred microliters of the media-diluted cell suspensions containing 10,000 cells/well were plated and incubated in 5% CO_2_ at 37 °C until confluence. The cells were treated with 2.5-, 5-, 10-, 20-, and 40-µM concentrations of luteolin, zinc oxide nanoparticles, and L-ZnONPs.

### 2.5. Measurement of Cell Viability Using MTT Assay

The MTT assay was performed as previously described by Denizot and Lang (1986) [57]. After 24 h, the treated cells were fixed using MTT reagent (5 mg/mL) in each well; cells were incubated at 37 °C for 1 h and centrifuged at 3000 rpm for 5 min. Plates were removed from centrifuge and the excess dye was washed, with 100 μL of DMSO added to solubilize the crystal. Optical density (OD) was taken at 570 nm, and percentage of inhibition was calculated using the formula mentioned below. The observations were represented graphically. Statistical one-way ANOVA analysis followed by Tukey’s test were conducted using the Prism 8 statistical analysis tool (GraphPad Software, San Diego, CA, USA).

% Inhibition = [(OD of control – OD of sample)/OD of control] × 100(1)

### 2.6. In Silico Anticancer Study

#### 2.6.1. Ligand Optimization Using Bioinformatics Software

**Figure 3:**
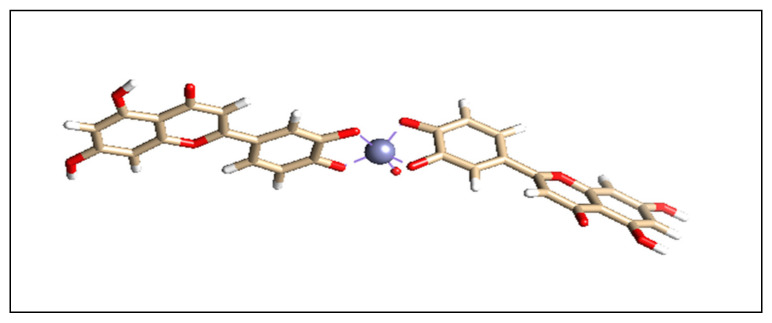
The 3D structure of the optimized L-ZnONPs.

Chemsketch 12.0 software was used to sketch the two-dimensional structure of the L-ZnONPs. After sketching, the structure was cleaned and the explicit hydrogens were added and saved in a file in .cml format for further use. For the molecular docking purpose we required the .pdb format file of the ligand, and hence the 2D .cml format file of the L-ZnONPs was converted to 3D .pdb format file by generating the 3D coordinates using OpenBabel v2.3.1 software [58] (http://openbabel.org/wiki/Main_Page, (accessed on 26 September 2016)). The obtained structure was further refined and geometrically cleaned using ArgusLab 4.0.1 software [59] (http://www.arguslab.com/arguslab.com/ArgusLab.html, (accessed on 26 September 2016)) At this point, the complex ligand molecule was geometrically fit for the molecular docking interaction studies (Figure 3).

#### 2.6.2. Protein Modeling, Validation, and Preparation Using Bioinformatics Software and Tools

Based on earlier reports, in this study six proteins (human polo-like kinase 1 (PDB Id: 1Q40), human protein kinase C (PDB Id: 2FK9), human HER2 kinase domain (PDB Id: 3PP0), human EGFR/HER3 kinase (PDB Id: 4RIW), human ataxia telangiectasia-mutated and Rad3-related (PDB Id: 5YZ0), and human vaccinia-related kinase 1 (PDB Id: 2LAV)), were considered for in silico validation of the mechanistic interactions responsible for L-ZnONP activity against cancer cells.

**Figure 4:**
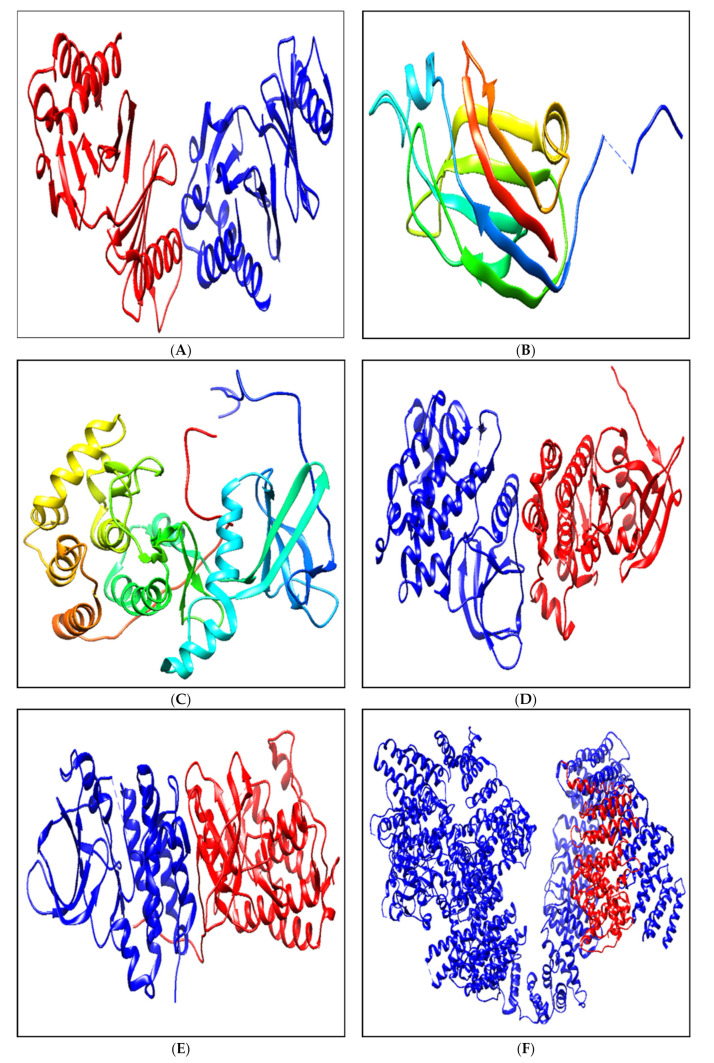
The ribbon 3D structure of the selected proteins (**A**) 1Q4O, (**B**) 2FK9, (**C**) 2LAV, (**D**) 3PP0, I 4RIW and (**F**) 5YZ0.

The 3D structures (.pdb format) of the above proteins were taken from the Protein Data Bank database (PDB) (https://www.rcsb.org/, (accessed on 28 November 2000)) depending on their resolution values (≥2Å) [60]. The downloaded .pdb format files of all the proteins were visualized using Chimera v1.3.7 software [61] (https://www.cgl.ucsf.edu/chimera/download.html, (accessed on 18 December 2020)) to edit the protein by deleting water and the other non-standard amino acids present with the protein (Figure 4A–F). The structures of the edited proteins were validated by the Ramachandran plot using RAMPAGE online tool in order to learn the number of residues in the favored and allowed regions [62]. The protein structure with ~96% of residues in the favored region and ~2 residues in the allowed region was selected for the molecular docking interaction purpose.

#### 2.6.3. Binding Site Residues

The active site pocket residues or the binding site residues where the ligand interacted with the protein molecule of all the selected proteins were obtained using the Galaxy web online tool http://galaxy.seoklab.org/cgi-bin/submit.cgi?type=REFINE, (accessed on 11 October 2020) [63].

#### 2.6.4. Molecular Interaction Studies

The validated proteins were now fit for the study of molecular interactions with the optimized ligand using the freely available and user-friendly PyRx 0.7 molecular docking software [64]. PyRx follows three main steps. Step one is to load the respective protein and L-ZnONP ligand to make the macromolecules and ligands to generate the .pdbqt files that have all of the required structural parameters for docking purposes. The second step is to select the binding site amino acid residues and build a grid box around the selected residues. In the final step, the docking process gets started by considering the genetic algorithm.

#### 2.6.5. Statistical Analysis

The results of anticancer activity are calculated as mean ± SE of three independent experiments. One-way analysis of variance (ANOVA) followed by Tukey’s multiple comparisons were carried out using the GraphPad Prism 8.0 statistical analysis software.

## 3. Results and Discussion

### 3.1. XRD Studies

**Figure 5:**
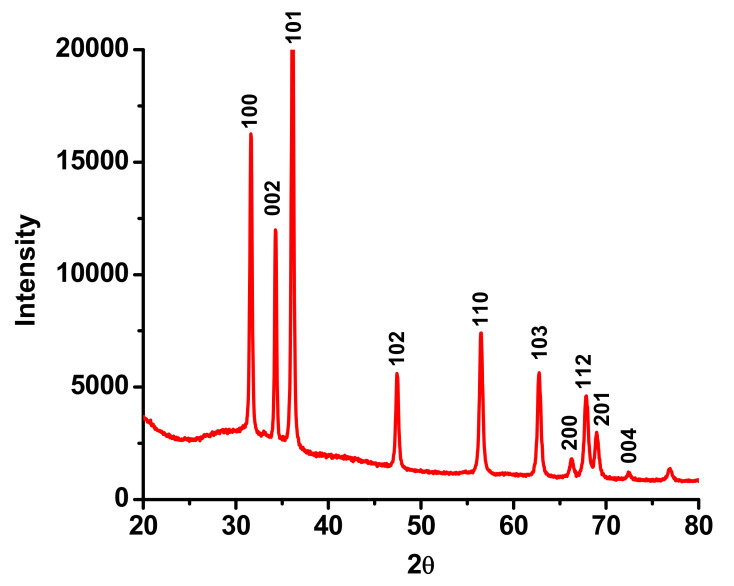
XRD diffraction pattern of as-synthesized L-ZnONPs.

The diffraction pattern was in accordance with the common ZnO hexagonal phase, i.e., the Wurtzite structure (JCPDS 36-1451) as manifested in Figure 5. The XRD pattern revealed the crystalline nature of as-obtained L-ZnONPs. The prominent diffraction peaks at angles (2θ) 31.98°, 34.53°, 36.28°, 47.68°, 56.54°, 62.94°, 66.52°, 67.94°, 69.12°, and 72.94° correspond to the reflections from the (100), (002), (101), (102), (110), (103), (200), (112), (201), and (004) planes, respectively.

### 3.2. SEM Analysis

**Figure 6:**
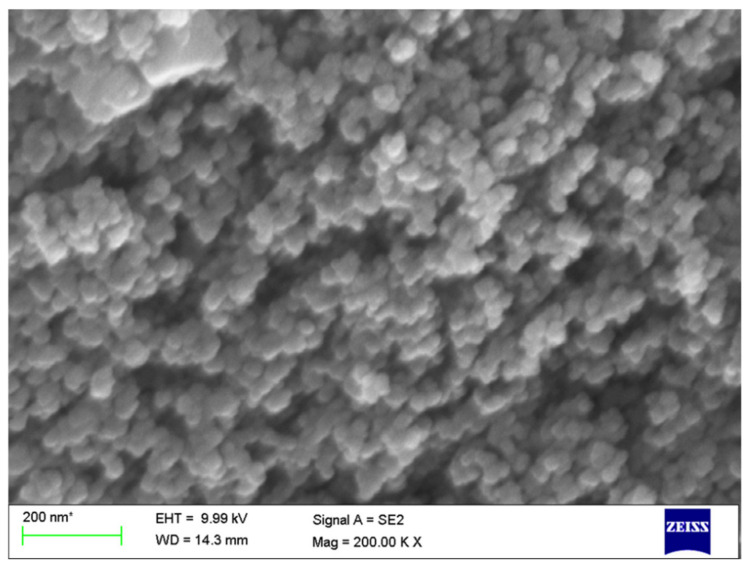
SEM images of as-obtained L-ZnONPs depicting nanospheres.

The morphology of L-ZnONPs under study was mainly composed of nanospheres and nanosheets with an average size ranging between 12 and 25 nm (Figure 6). It can be clearly seen from this Figure that the morphology is comprised of dense cloud with randomly oriented, overlapping nanosheets and cluttered nanospheres.

### 3.3. TEM Analysis

**Figure 7:**
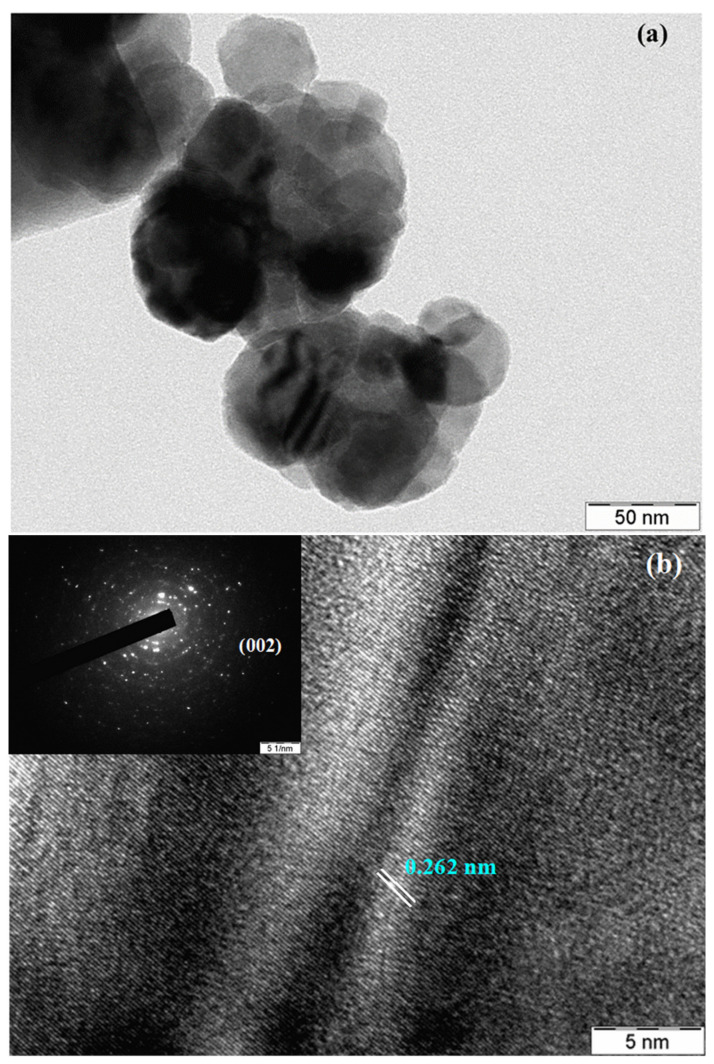
(**a**) TEM and (**b**) HR-TEM micrographs with SAED patterns (inset) of as-synthesized L-ZnONPs.

The size and morphology of the as-obtained L-ZnONPs was further confirmed by TEM studies. As shown in Figure 7a, the TEM analysis of L-ZnONPs confirms that the particles reported here were almost hexagonal with particle size of approximately 17 nm, which is consistent with the observed morphology in SEM investigations. In addition, we can also observe spherical and rod-shaped nanostructures with some agglomerations of larger and smaller particles. The crystallinity results obtained by XRD analysis were further supported by HRTEM studies. The diffraction lattice fringes (Figure 7b) in the obtained L-ZnONPs show the d-spacing between two lattice fringes. In our case, the inter-planar distance between the two fringes was 0.262 nm, which corresponds with the d-spacing of the (002) crystal plane of ZnO [12].

### 3.4. L-ZnONPs Showed Greater Cytotoxicity in the MCF-7 Cell Line

**Figure 8:**
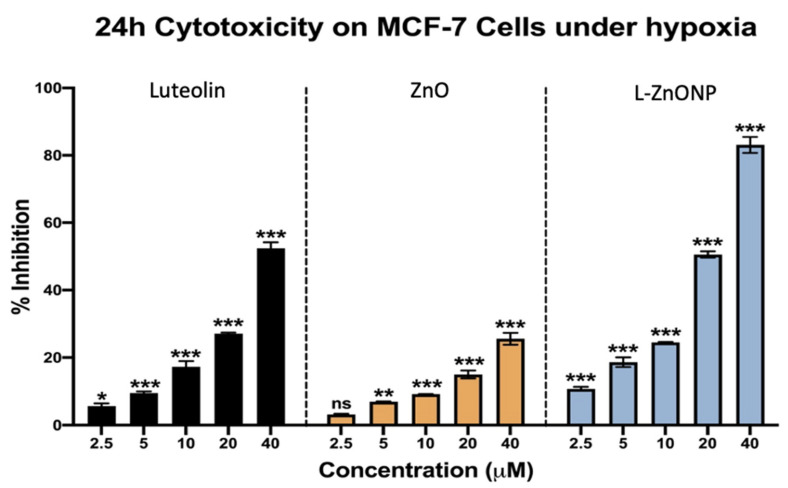
Anticancer activity of luteolin, ZnO, and L-ZnONPs on MCF-7 cell lines. * p < 0.033, ** p < 0.002, *** p < 0.001, ns = not significant.

All the treatment groups (luteolin, ZnO, and L-ZnONPs) showed dose-dependent cytotoxicity in MCF-7. However, the cytotoxicity in the treatment group containing the nanoparticle-coated luteolin was significantly greater than that of the individual treatments of the compound and ZnO. The cells were treated in hypoxic conditions for 24 h, with differential concentrations of the test samples ranging from 2.5 to 40 μM. While the highest concentration of luteolin showed cell growth inhibition of ~52%, the MCF-7 cell growth in the ZnO nanoparticle-treated group was inhibited by up to ~25% with a similar dosage (Figure 8).

Surprisingly, the anti-breast cancer potential against the MCF-7 cells was evidently greater with L-ZnONP treatment at a 40-μM concentration, with the number of viable cells reduced to a paltry 15%. Luteolin has been reported to have an IC_50_ value of about 43 μM for the breast cancer cell line MCF-7 [35]. Furthermore, the nanoparticle delivery of luteolin was found to reduce its IC_50_ value in both in vitro and in vivo models [65], thereby suggesting that ZnO nanoparticle-guided delivery of luteolin improves its anti-tumorigenic activity.

### 3.5. In Silico Protein Validation

In this study, all the selected proteins showed over 96% of residues in the favored region and more than 2% of residues in the allowed region; thus, the RAMPAGE results were significant for considering proteins for further molecular interactions (Table 1).

### 3.6. Molecular Docking Studies

After the docking process, the six docked poses of the L-ZnONP system against the particular protein were obtained based on the increasing value of the binding affinity. The pose with the least binding affinity was selected and its .pdb format file was saved.

The docked ligand result file with the protein .pdb was visualized using Pymol 1.4.1 software https://pymol.org/2/, (accessed on 25 January 2021) to check for bonded and non-bonded interactions between the ligand and the protein [66].

### 3.7. Interaction of L-ZnONPs with Proteins

**Figure 9:**
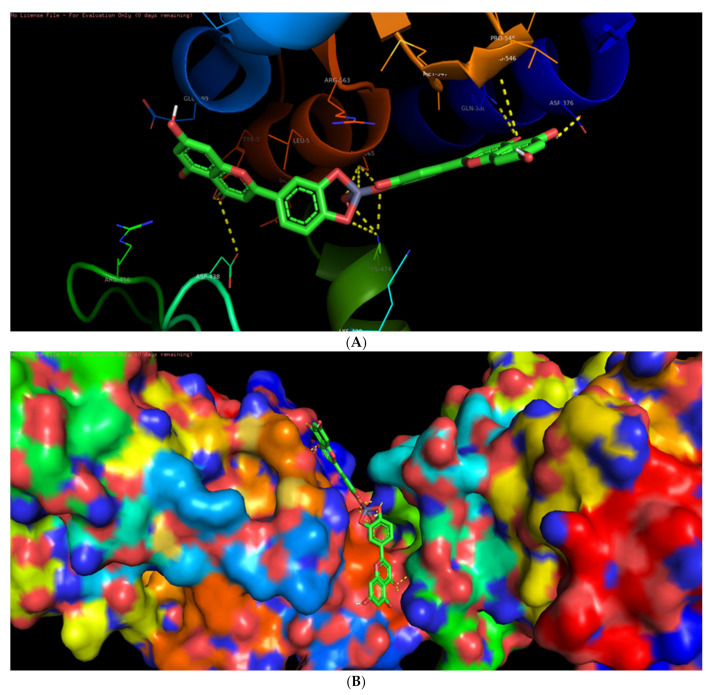
(**A**) The bonded and non-bonded interactions of L-ZnONPs with the cancer protein 1Q4O. (**B**) The best-docked pose of L-ZnONPs bound to the hydrophobic preset of the 1Q4O protein.

**Figure 10:**
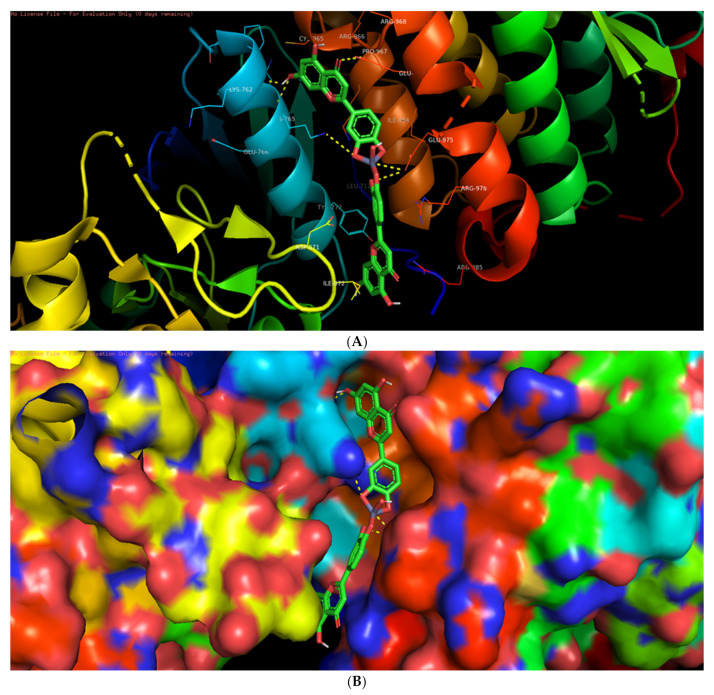
(**A**) The bonded and non-bonded interactions of L-ZnONPs with the cancer protein 3PP0. (**B**) The best-docked pose of L-ZnONPs bound to the hydrophobic preset of the 3PP0 protein.

**Figure 11:**
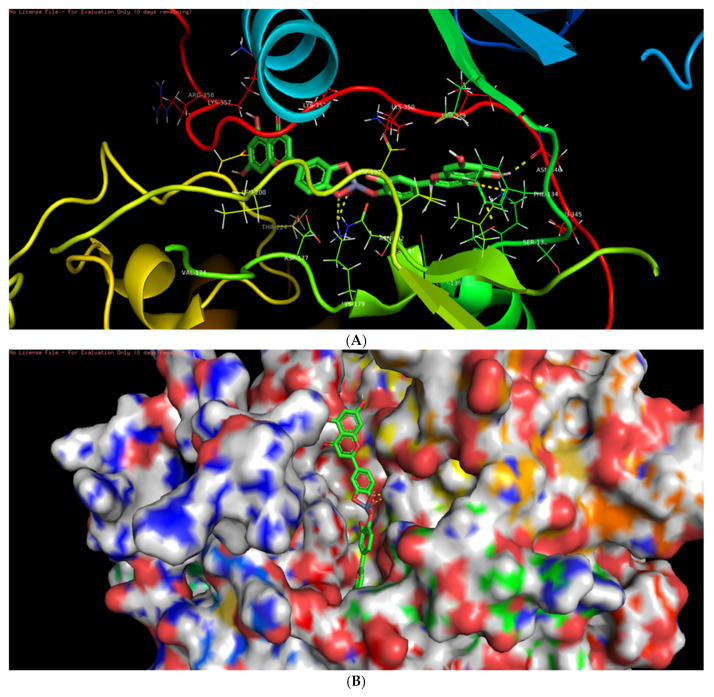
(**A**) The bonded and non-bonded interactions of L-ZnONPs with the cancer protein 2LAV. (**B**) The best-docked pose of L-ZnONPs bound to the hydrophobic preset of the 2LAV protein.

**Figure 12:**
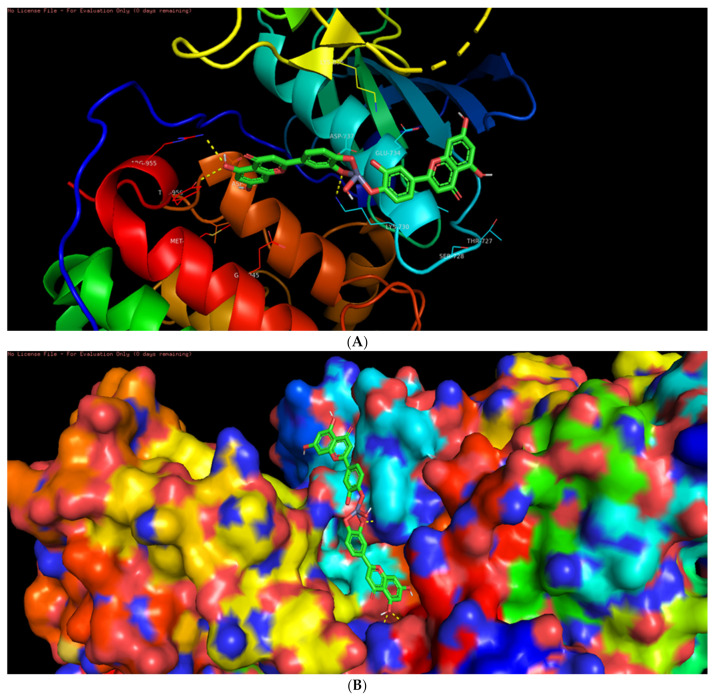
(**A**) The bonded and non-bonded interactions of L-ZnONPs with the cancer protein 4RIW. (**B**) The best-docked pose of L-ZnONPs bound to the hydrophobic preset of the 4RIW protein.

**Figure 13:**
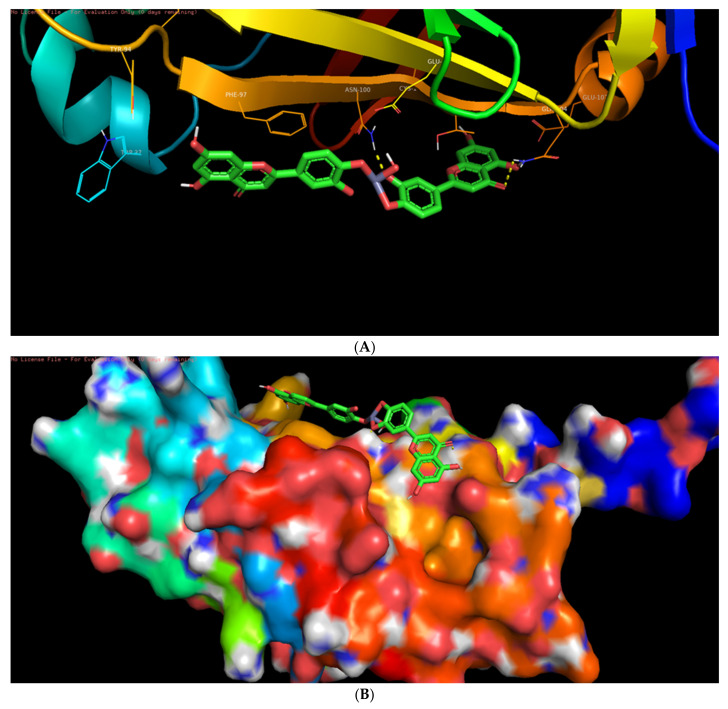
(**A**) The bonded and non-bonded interactions of L-ZnONPs with the cancer protein 2FK9. (**B**) The best-docked pose of L-ZnONPs bound to the hydrophobic preset of the 2FK9 protein.

**Figure 14:**
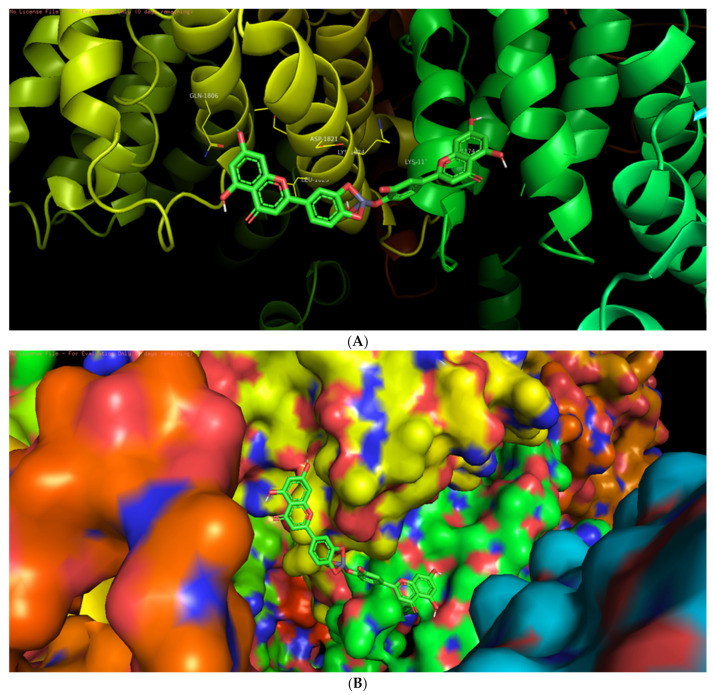
(**A**) The bonded and non-bonded interactions of L-ZnONPs with the cancer protein 5YZ0. (**B**) The best-docked pose of L-ZnONPs bound to the hydrophobic preset of the 5YZ0 protein.

Among the six selected proteins, L-ZnONPs showed significant interactions with 1Q4O, 3PP0, and 2LAV by forming 11, 6, and 5 hydrogen bonds with values of −9.7, −8.3 and −10.1 for binding affinity, respectively (Figure 9A, Figure 10, Figure 11, Figure 12, Figure 13 and Figure 14B). The other three proteins 4RIW, 2FK9, and 5YZ0 showed comparatively less i.e., 3 (−5.2), 2 (−7.6) and no (−7.5) hydrogen bonding with the L-ZnONPs, respectively. The best docked poses of the ligand with the selected six proteins were found to have conserved salt bridges with large numbers of bonded and non-bonded interactions (Figure S5).

Based on the above results, it was hypothesized that the L-ZnONPs inhibited the MCF-7 cell proliferation by the means of molecular interactions involving the human polo-like kinase 1 (PLK1) protein [34,42].

## 4. Conclusions

In the present study, we obtained zinc oxide nanoparticles with a convenient green approach using a phyto-molecule, luteolin, isolated from *Eclipta alba.* The analysis of the as-obtained L-ZnONPs by electron microscope studies revealed that the prepared material assumed a hexagonal shape with particle size of approximately 17 nm. The d-spacing between two lattice fringes was shown to be 0.262 nm, corresponding with the d-spacing of the (002) crystal plane of ZnO. The luteolin-capped ZnONPs showed better tumoricidal behavior as compared to the two other components when tested individually. Based on in silico observations it may be hypothesized that the MCF-7 cytotoxicity of L-ZnONPs occurs via the involvement of the PLK1 proteins.

## Figures and Tables

**Table 1:** The RAMPAGE results for all the selected proteins.

Sl No.	Protein PDB ID	Favored Region %	Allowed Region %	Outlier Region %
1	1Q4O	96.6	2.7	0.7
2	2FK9	96.6	3.4	0.0
3	2LAV	98.7	1.3	0.0
4	3PP0	97.8	2.2	0.0
5	4RIW	98.5	1.5	0.0
6	5YZ0	98.1	1.9	0.0

## Data Availability

Not applicable.

## Supplementary Materials

The following are available online at https://www.mdpi.com/2218-273X/11/3/385/s1. Figure S1: Proton NMR of Luteolin molecule, Figure S2: Mass spectrum of Luteolin molecule depicting the molecular ion peak at m/z = 286.3073, Figure S3: FT-IR spectra of (a) Luteolin and (b) as-prepared L-ZnONPs, Figure S4: UV-Visible spectrum of the as-obtained L-ZnONPs, Figure S5: The after docking 3D structures of L-ZnONPs with (A) 1Q4O, (B) 2FK9, (C) 2LAV, (D) 3PP0, (E) 4RIW and (F) 5YZ0.

## Abbreviations

AKT	Protein kinase B
DMEM	Dulbecco’s Modified Eagle Medium
DR5	Death receptor 5
EGFR/HER	Endothelial growth factor receptor
ER	Estrogen receptor
ESP	Estrogen signaling pathway
ERK	Extracellular signal-regulated kinase
JNK	Jun N-terminal kinase
MAPK	Mitogen-activated protein kinase
NF-kB	Nuclear factor kappa B
PARP	Poly ADP-ribose polymerase
PI3K	Phosphatidylinositol 3-kinase
PKC	Protein kinase C
PLK-1	Polo-like kinase 1
PR	Progesterone receptor
ROS	Reactive oxygen species
TNFa	Tumor necrosis factor alpha
TLP2	Tumor progression locus 2
VEGF	Vascular endothelial growth factor
