# Supplementary material for: Luteolin-Fabricated ZnO Nanostructures Showed PLK-1 Mediated Anti-Breast Cancer Activity
Source: Biomolecules. 2021 Mar 5;11(3):385. doi: 10.3390/biom11030385 (PMC7998981; doi:10.3390/biom11030385)
Supplement: Supplementary file 1 [file biomolecules-11-00385-s001.pdf]

Supporting information

Luteolin-Fabricated ZnO Nanostructures Showed PLK-1 Mediated Anti-breast Cancer Activity

Shiva Prasad Kollur [1,*], Shashanka K. Prasad [2], Sushma Pradeep [2], Ravindra Veerapur [3], Sharanagouda S. Patil [4], Raghavendra G. Amachawadi [5], Rajendra Prasad S [6], Ghada Lamraoui [7], Abdulaziz A. Al-Kheraif [8], Abdallah M. Elgorban [9], Asad Syed [9,*] and Chandan Shivamallu [2,*]

[1] Department of Sciences, Amrita School of Arts and Sciences, Amrita Vishwa Vidyapeetham, Mysuru Campus, Mysuru, Karnataka–570 026, India.

[2] Department of Biotechnology and Bioinformatics, School of Life Sciences, JSS Academy of Higher Education and Research, Mysuru, Karnataka–570 015, India; shashankaprasad@jssuni.edu.in (S.K.P); sushmap@jssuni.edu.in (S.P)

[3] Department of Metallurgy and Materials Engineering, Malawi Institute of Technology, Malawi University of Science and Technology, P.O . Box - 5916, Limbe, Malawi; rveerapur@must.ac.mw (R.V)

[4] ICAR-National Institute of Veterinary Epidemiology and Disease Informatics, Yelahanka, Bengaluru, Karnataka, India. sharanspin13@gmail.com (S.P)

[5] Department of Clinical Sciences, College of Veterinary Medicine, Kansas State University, Manhattan, KS, United States of America. agraghav@vet.k-state.edu (R.G.A)

[6] Department of Chemistry, Davangere University, Shivagangotri, Davangere, Karnataka 577 007, India; raju.rajendraprasad693@gmail.com (R.P.S)

[7] Nature and Life Sciences, Earth and Universe Sciences, University of Tlemcen, Tlemcen, Algeria; lamraouig@gmail.com (G.L)

[8] Dental Biomaterials Research Chair, Dental Health Department, College of Applied Medical Sciences, King Saud University, P.O. Box 10219, Riyadh 11433, Saudi Arabia. aalkhuraif@ksu.edu.sa (A.A.A.K)

[9] Department of Botany and Microbiology, College of Science, King Saud University, P.O. Box 2455, Riyadh 11451, Saudi Arabia. aelgorban@ksu.edu.sa (A.M.E)

* Correspondence: shivachemist@gmail.com (K.S.P); assyed@ksu.edu.sa (A.S); chandans@jssuni.edu.in (C.S.)

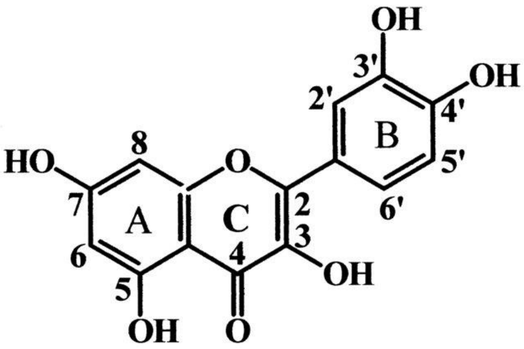

Structure of Luteolin

Characterization: Pale yellow needles; m.p. 325-328 °C; ESI-MS (TOF) *m*/z = 286.3073 $[M]^+$; ^{1}H-NMR (DMSO-d_6, 300 MHz, ppm)- δ = 7.29-7.79 (5H, multiplet), 10.39 (4H, s). The structure was confirmed by comparison with literature data [Hao Liu, Yan Mou, Jianglin Zhao, Jihua Wang, Ligang Zhou, Mingan Wang, Daoquan Wang, Jianguo Han, Zhu Yu and Fuyu Yang. Flavonoids from *Halostachys caspica* and Their Antimicrobial and Antioxidant Activities. *Molecules* 2010, *15*, 7933-7945; doi: 10.3390/molecules15117933].

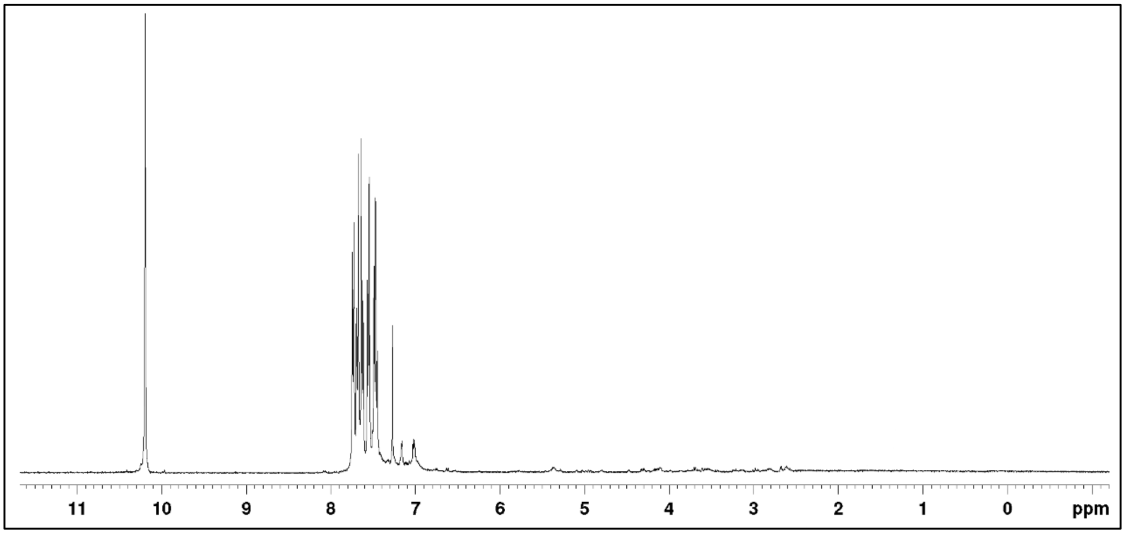

Figure S1. Proton NMR of Luteolin molecule.

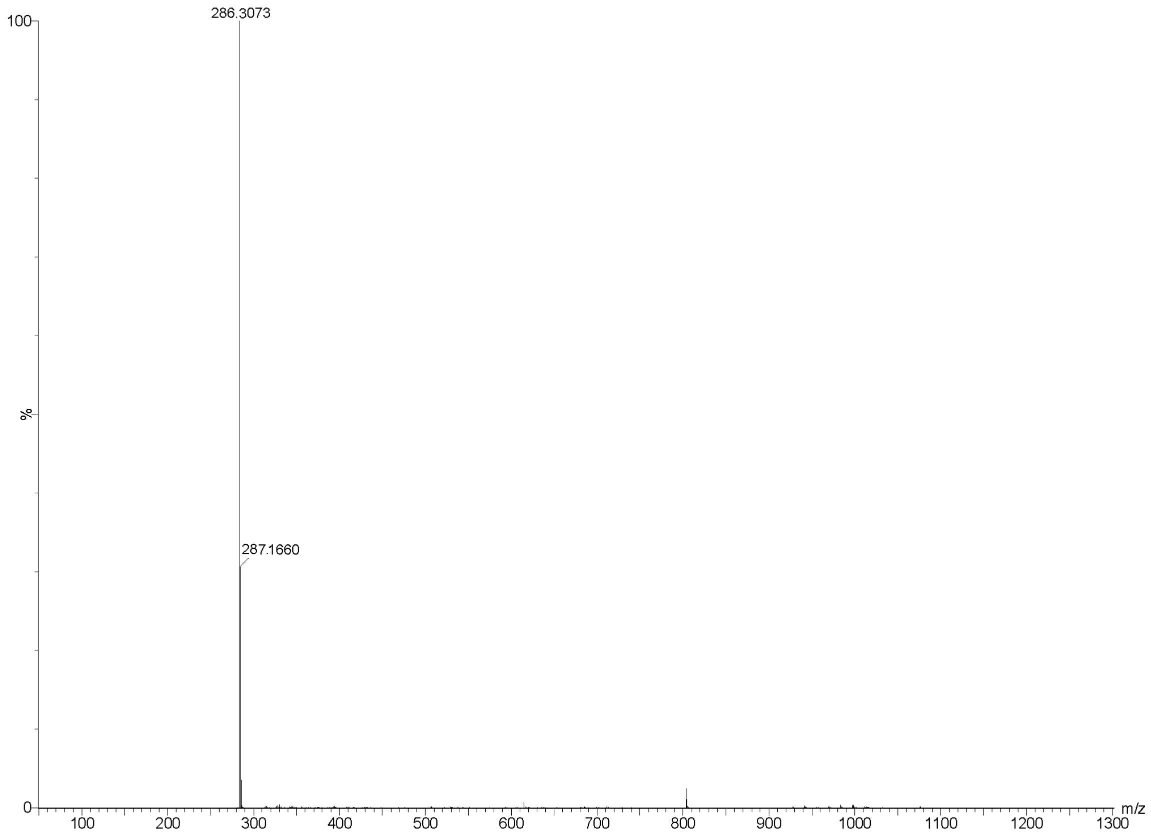

Figure S2. Mass spectrum of Luteolin molecule depicting the molecular ion peak at m/z = 286.3073.

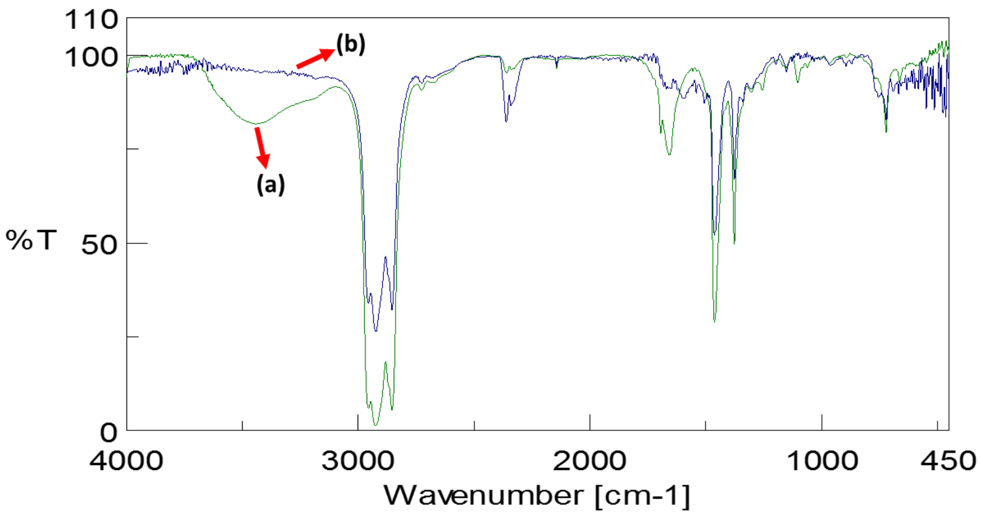

Figure S3. FT-IR spectra of (a) Luteolin and (b) as-prepared L-ZnONPs.

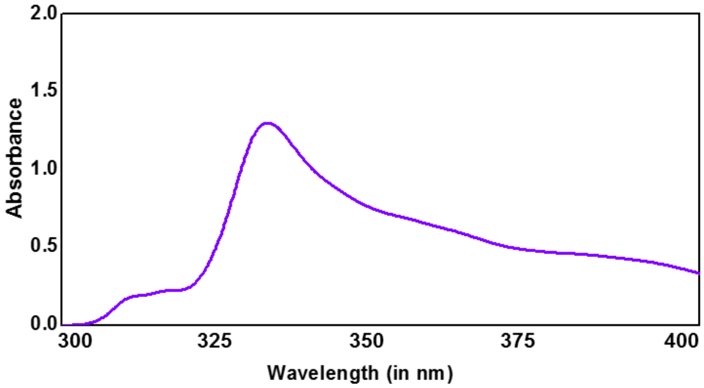

Figure S4. UV-Visible spectrum of the as-obtained L-ZnONPs.

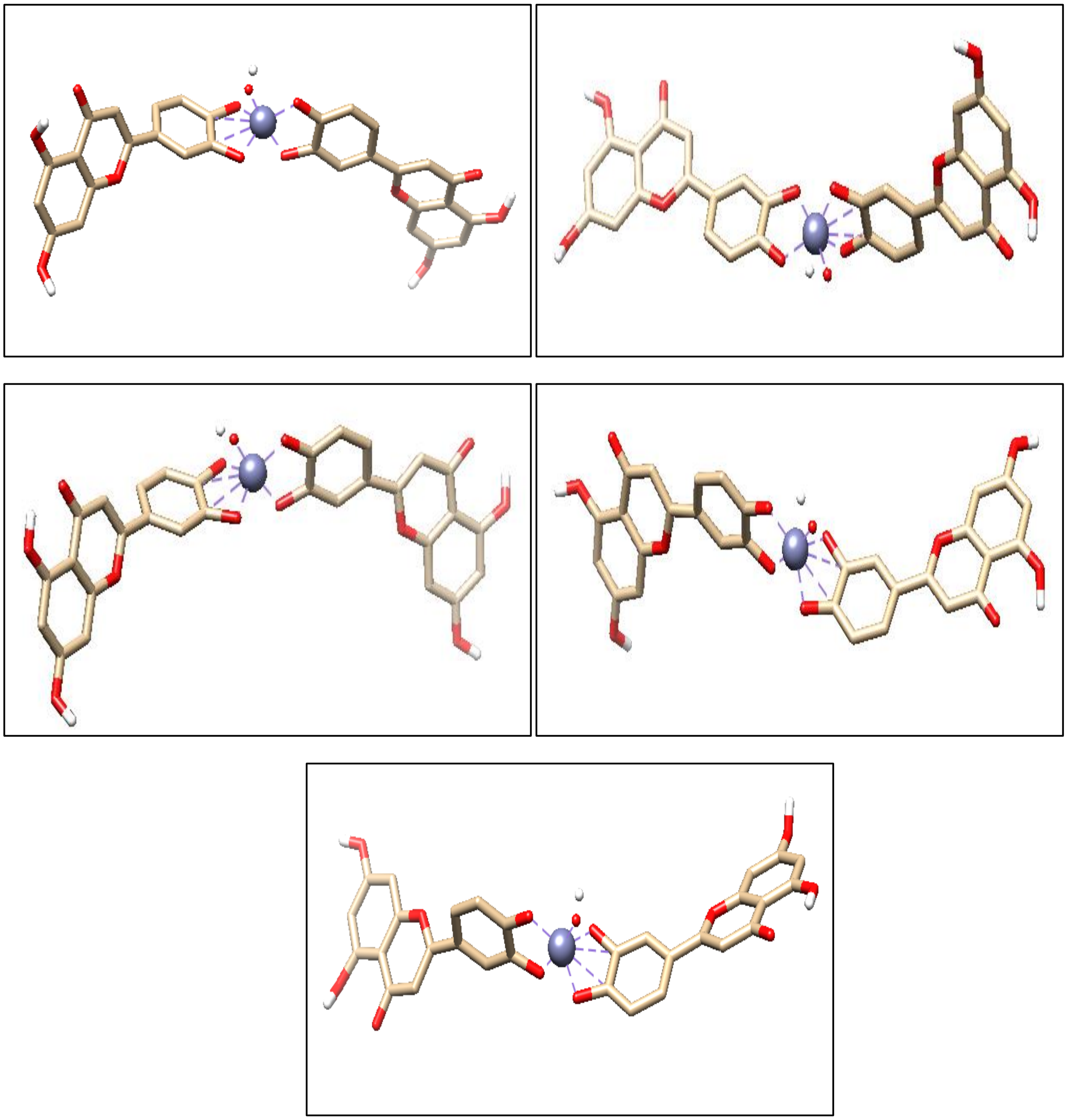

Figure S5. The after docking 3D structures of L-ZnONPs with A) 1Q4O, B) 2FK9, C) 2LAV, D) 3PP0, E) 4RIW and F) 5YZ0.
